# (*R*)-1-Phenyl­ethanaminium (*S*)-4-chloro­mandelate

**DOI:** 10.1107/S1600536808003516

**Published:** 2008-02-06

**Authors:** Quan He, Michael C. Jennings, Sohrab Rohani, Jesse Zhu, Hassan Gomaa

**Affiliations:** a Department of Chemical Engineering, Ningbo University of Technology, Ningbo 315016, People’s Republic of China; b Department of Chemistry, University of Western Ontario, London, Ontario, N6A 5B7, Canada; c Department of Chemical and Biochemical Engineering, University of Western Ontario, London, Ontario, N6A 5B9, Canada

## Abstract

The absolute configuration of the title complex, C_8_H_12_N^+^·C_8_H_6_ClO_3_^−^ or [*R*-C_6_H_5_C(H)CH_3_NH_3_][*S*-4-ClC_6_H_4_C(H)(OH)CO_2_], has been confirmed by the structure determination. In the crystal structure, inter­molecular O—H⋯O and N—H⋯O hydrogen bonds form a two-dimensional network perpendicular to the *c* axis.

## Related literature

For background information and the crystal structure of the *R*,*R* diastereomer of the title compound, see: He *et al.* (2007 ▶).
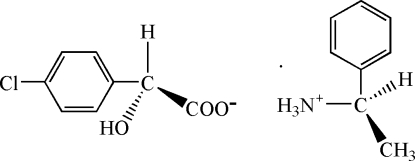


## Experimental

### 

#### Crystal data

C_8_H_12_N^+^·C_8_H_6_ClO_3_^−^
*M* *_r_* = 307.76
Monoclinic, 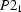

*a* = 10.4091 (7) Å
*b* = 5.7635 (4) Å
*c* = 13.2544 (10) Å
β = 96.831 (4)°
*V* = 789.52 (10) Å^3^
*Z* = 2
Mo *K*α radiation
μ = 0.25 mm^−1^
*T* = 150 (2) K
0.45 × 0.15 × 0.08 mm

#### Data collection

Nonius KappaCCD diffractometer
Absorption correction: multi-scan (*SORTAV*; Blessing, 1995 ▶) *T*_min_ = 0.787, *T*_max_ = 0.984
7943 measured reflections
3431 independent reflections
2881 reflections with *I* > 2σ(*I*)
*R*_int_ = 0.072

#### Refinement

*R*[*F* ^2^ > 2σ(*F* ^2^)] = 0.044
*wR*(*F* ^2^) = 0.115
*S* = 1.05
3431 reflections
192 parameters
1 restraint
H-atom parameters constrained
Δρ_max_ = 0.26 e Å^−3^
Δρ_min_ = −0.30 e Å^−3^
Absolute structure: Flack (1983 ▶), 1436 Friedel pairs
Flack parameter: −0.03 (8)

### 

Data collection: *COLLECT* (Nonius, 1997 ▶); cell refinement: *DENZO-SMN* (Otwinowski & Minor, 1997 ▶); data reduction: *DENZO-SMN*; program(s) used to solve structure: *SHELXS97* (Sheldrick, 2008 ▶); program(s) used to refine structure: *SHELXL97* (Sheldrick, 2008 ▶); molecular graphics: *SHELXTL/PC* (Sheldrick, 2008 ▶); software used to prepare material for publication: *SHELXTL/PC*.

## Supplementary Material

Crystal structure: contains datablocks global, I. DOI: 10.1107/S1600536808003516/lh2591sup1.cif

Structure factors: contains datablocks I. DOI: 10.1107/S1600536808003516/lh2591Isup2.hkl

Additional supplementary materials:  crystallographic information; 3D view; checkCIF report

## Figures and Tables

**Table 1:** Hydrogen-bond geometry (Å, °)

*D*—H⋯*A*	*D*—H	H⋯*A*	*D*⋯*A*	*D*—H⋯*A*
O9—H9*A*⋯O9^i^	0.84	2.26	2.939 (2)	139
O9—H9*A*⋯O11^i^	0.84	2.09	2.826 (2)	146
N13—H13*A*⋯O12^ii^	0.91	1.89	2.798 (2)	172
N13—H13*B*⋯O11	0.91	1.83	2.731 (2)	169
N13—H13*C*⋯O12^iii^	0.91	1.88	2.779 (2)	171

Symmetry codes: (i) 
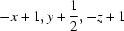
; (ii) 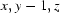; (iii) 
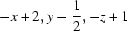
.

**Fig. 1.**
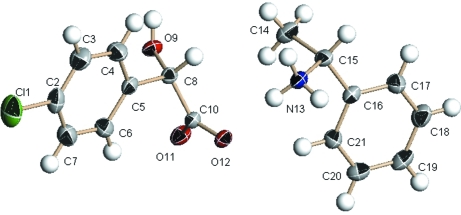
The molecular structure of the title compound with 30% probability displacement ellipsoids and the atom labelling scheme.

## supplementary crystallographic information

### Comment

In our previous work, phenylethylamine (PEA) has been proven to be an efficient resolving agent for resolution of racemic 4-chloromandelic acid. In order to further investigate the chiral recognition mechanism, the single-crystal structure of the corresponding more soluble salt, (*R*)-Phenylethylammonium (*S*)-4-chloromandelate, is reported here for comparison with that of the less soluble salt (*R*)-Phenylethylammonium (*R*)-4-chloromandelate (He *et al.*, 2007).

The title complex consists of an ion pair; an amine cation and a carboxylate anion (see Fig. 1). The absolute stereochemistry of each ion has been confirmed by the structure determination [absolute structure parameter -0.03 (8); (Flack, 1983)]. All three H atoms of the –NH_3_ group and the H atom of the O—H group act as hydrogen bond donors in intermolecular O—H···O and N—H···O hydrogen bonds, forming a two-dimensional network perpendicular to the *c* axis. The hydrogen bond motif in the title compound is different to that observed in the room temperature structure of the *R*,*R* diastereomer (He *et al.*, 2007).

### Experimental

To a solution (*S*)-4-chloromandelate (2.0 g, 0.01 mol) in 10 ml 2-propanol, (1.3 mL, 0.01 mol), (*R*)-Phenylethylammonium, was added gradually. A white crystalline solid appeared. The crystals were collected and washed with 2-propanol twice to give the title compound (1.85 g), yield 56%. Single crystals were grown from a concentrated methanol solution of the title compound by slow evaporation at room temperature.

### Refinement

All H atoms were positioned geometrically and constrained as riding atoms with; C—H = 1.00Å and *U*_iso_(H) = 1.2*U*_eq_(C) for methyne H atoms, C—H = 0.98Å and *U*_iso_(H) = 1.5*U*_eq_(C) for methyl H atoms, C—H = 0.95Å and *U*_iso_(H) = 1.2*U*_eq_(C) for aromatic H atoms, O—H = 0.84Å and *U*_iso_(H) = 1.5*U*_eq_(C) for hydroxyl H atoms and N—H = 0.91Å and *U*_iso_(H) = 1.5*U*_eq_(C) for amine H atoms.

### Crystal data

**Table:**

C_8_H_12_N^+^·C_8_H_6_ClO_3_^–^	*F*_000_ = 324
*M**_r_* = 307.76	*D*_x_ = 1.295 Mg m^−^^3^
Monoclinic, *P*2_1_	Mo *K*α radiation λ = 0.71073 Å
Hall symbol: P 2yb	Cell parameters from 11634 reflections
*a* = 10.4091 (7) Å	θ = 2.0–27.5º
*b* = 5.7635 (4) Å	µ = 0.25 mm^−^^1^
*c* = 13.2544 (10) Å	*T* = 150 (2) K
β = 96.831 (4)º	Plate, colourless
*V* = 789.52 (10) Å^3^	0.45 × 0.15 × 0.08 mm
*Z* = 2	

### Data collection

**Table:**

Nonius KappaCCD diffractometer	3431 independent reflections
Radiation source: fine-focus sealed tube	2881 reflections with *I* > 2σ(*I*)
Monochromator: graphite	*R*_int_ = 0.072
*T* = 150(2) K	θ_max_ = 27.5º
φ scans, and ω scans with κ offsets	θ_min_ = 2.4º
Absorption correction: multi-scan(SORTAV; Blessing, 1995)	*h* = −13→13
*T*_min_ = 0.787, *T*_max_ = 0.984	*k* = −7→7
7943 measured reflections	*l* = −16→17

### Refinement

**Table:**

Refinement on *F*^2^	Hydrogen site location: inferred from neighbouring sites
Least-squares matrix: full	H-atom parameters constrained
*R*[*F*^2^ > 2σ(*F*^2^)] = 0.044	*w* = 1/[σ^2^(*F*_o_^2^) + (0.0565*P*)^2^ + 0.1172*P*] where *P* = (*F*_o_^2^ + 2*F*_c_^2^)/3
*wR*(*F*^2^) = 0.115	(Δ/σ)_max_ < 0.001
*S* = 1.05	Δρ_max_ = 0.26 e Å^−^^3^
3431 reflections	Δρ_min_ = −0.30 e Å^−^^3^
192 parameters	Extinction correction: none
1 restraint	Absolute structure: Flack (1983), 1436 Fridel pairs
Primary atom site location: structure-invariant direct methods	Flack parameter: −0.03 (8)
Secondary atom site location: difference Fourier map	

### Special details

**Table:**

Experimental. Absorption correction: multi-scan from symmetry-related measurements (*SORTAV*; Blessing, 1995). *M*.p. 140.8–142.5 K. The specific rotation was [α]^25^_D_ = +50.5° (c=1, C^1^H^3^OH), determined using a Perkin Elmer Model 341 Digital Polarimeter; ^1^H-NMR (d_6_-DMSO/TMS): δ 1.424 (d, 3H, CH_3_), 4.300 (m, 1H, CHNH_2_), 4.536 (s, 1H, CHOH), 7.268–7.457 (m, 9H, C_6_H_5_ and C_6_H_4_Cl) measured using an INOVA 400 MHz NMR (Varian).
Geometry. All e.s.d.'s (except the e.s.d. in the dihedral angle between two l.s. planes) are estimated using the full covariance matrix. The cell e.s.d.'s are taken into account individually in the estimation of e.s.d.'s in distances, angles and torsion angles; correlations between e.s.d.'s in cell parameters are only used when they are defined by crystal symmetry. An approximate (isotropic) treatment of cell e.s.d.'s is used for estimating e.s.d.'s involving l.s. planes.
Refinement. Refinement of *F*^2^ against ALL reflections. The weighted *R*-factor *wR* and goodness of fit *S* are based on *F*^2^, conventional *R*-factors *R* are based on *F*, with *F* set to zero for negative *F*^2^. The threshold expression of *F*^2^ > σ(*F*^2^) is used only for calculating *R*-factors(gt) *etc*. and is not relevant to the choice of reflections for refinement. *R*-factors based on *F*^2^ are statistically about twice as large as those based on *F*, and *R*- factors based on ALL data will be even larger.

### Fractional atomic coordinates and isotropic or equivalent isotropic displacement parameters (Å^2^)

**Table:**

	*x*	*y*	*z*	*U*_iso_*/*U*_eq_	
Cl1	0.62566 (7)	0.61666 (16)	1.01588 (5)	0.0611 (3)	
C2	0.6271 (2)	0.5894 (5)	0.88508 (17)	0.0393 (6)	
C3	0.5788 (3)	0.7663 (5)	0.82206 (19)	0.0435 (6)	
H3A	0.5408	0.8990	0.8490	0.052*	
C4	0.5863 (2)	0.7484 (4)	0.71887 (18)	0.0368 (5)	
H4A	0.5529	0.8702	0.6751	0.044*	
C5	0.64141 (18)	0.5567 (4)	0.67818 (16)	0.0260 (4)	
C6	0.6887 (2)	0.3804 (4)	0.74326 (19)	0.0359 (5)	
H6A	0.7267	0.2473	0.7165	0.043*	
C7	0.6815 (2)	0.3952 (5)	0.84688 (19)	0.0422 (6)	
H7A	0.7137	0.2730	0.8909	0.051*	
C8	0.64285 (18)	0.5435 (3)	0.56394 (15)	0.0246 (4)	
H8A	0.6574	0.7033	0.5382	0.030*	
O9	0.52152 (12)	0.4619 (3)	0.51580 (11)	0.0287 (3)	
H9A	0.4701	0.5740	0.5053	0.043*	
C10	0.74941 (19)	0.3868 (4)	0.53386 (15)	0.0249 (4)	
O11	0.72224 (14)	0.1839 (3)	0.50678 (13)	0.0345 (4)	
O12	0.86102 (13)	0.4771 (3)	0.53936 (11)	0.0299 (3)	
N13	0.87681 (16)	−0.1239 (3)	0.42117 (14)	0.0269 (4)	
H13A	0.8657	−0.2587	0.4548	0.040*	
H13B	0.8332	−0.0076	0.4487	0.040*	
H13C	0.9625	−0.0885	0.4267	0.040*	
C14	0.6876 (2)	−0.2471 (5)	0.30409 (19)	0.0395 (6)	
H14A	0.6898	−0.4075	0.3288	0.059*	
H14B	0.6480	−0.2430	0.2332	0.059*	
H14C	0.6366	−0.1517	0.3458	0.059*	
C15	0.8256 (2)	−0.1521 (4)	0.31099 (16)	0.0296 (5)	
H15A	0.8223	0.0045	0.2783	0.035*	
C16	0.91365 (19)	−0.3052 (4)	0.25701 (17)	0.0278 (5)	
C17	0.9388 (2)	−0.2491 (4)	0.15998 (17)	0.0348 (5)	
H17A	0.9041	−0.1103	0.1292	0.042*	
C18	1.0142 (2)	−0.3926 (5)	0.10662 (18)	0.0425 (6)	
H18A	1.0293	−0.3536	0.0394	0.051*	
C19	1.0672 (2)	−0.5923 (5)	0.15193 (19)	0.0400 (6)	
H19A	1.1194	−0.6903	0.1160	0.048*	
C20	1.0443 (2)	−0.6496 (4)	0.24955 (19)	0.0376 (6)	
H20A	1.0815	−0.7859	0.2810	0.045*	
C21	0.9671 (2)	−0.5080 (4)	0.30114 (17)	0.0325 (5)	
H21A	0.9502	−0.5495	0.3677	0.039*	

### Atomic displacement parameters (Å^2^)

**Table:**

	*U*^11^	*U*^22^	*U*^33^	*U*^12^	*U*^13^	*U*^23^
Cl1	0.0659 (5)	0.0910 (7)	0.0280 (3)	−0.0131 (4)	0.0120 (3)	−0.0052 (4)
C2	0.0348 (12)	0.0566 (15)	0.0273 (11)	−0.0126 (12)	0.0076 (9)	−0.0033 (12)
C3	0.0474 (15)	0.0465 (15)	0.0389 (14)	0.0067 (12)	0.0141 (11)	−0.0070 (13)
C4	0.0438 (13)	0.0333 (12)	0.0342 (12)	0.0085 (11)	0.0089 (10)	−0.0005 (10)
C5	0.0202 (9)	0.0275 (11)	0.0311 (10)	−0.0025 (8)	0.0058 (8)	−0.0028 (9)
C6	0.0376 (12)	0.0329 (11)	0.0383 (13)	0.0040 (10)	0.0094 (10)	0.0050 (11)
C7	0.0415 (13)	0.0485 (15)	0.0366 (13)	0.0003 (12)	0.0048 (10)	0.0083 (12)
C8	0.0202 (9)	0.0239 (10)	0.0299 (11)	−0.0023 (8)	0.0035 (8)	−0.0004 (8)
O9	0.0199 (7)	0.0278 (8)	0.0376 (9)	0.0015 (6)	0.0000 (6)	−0.0031 (7)
C10	0.0223 (9)	0.0273 (11)	0.0256 (10)	0.0011 (8)	0.0052 (8)	0.0004 (9)
O11	0.0290 (8)	0.0286 (8)	0.0474 (10)	−0.0002 (6)	0.0104 (7)	−0.0071 (7)
O12	0.0208 (7)	0.0323 (8)	0.0373 (8)	−0.0001 (6)	0.0067 (6)	0.0026 (7)
N13	0.0233 (8)	0.0258 (9)	0.0325 (9)	0.0003 (7)	0.0066 (7)	−0.0027 (8)
C14	0.0272 (11)	0.0549 (15)	0.0361 (13)	0.0034 (11)	0.0029 (9)	−0.0099 (12)
C15	0.0290 (11)	0.0321 (12)	0.0284 (11)	0.0066 (9)	0.0063 (9)	0.0001 (9)
C16	0.0226 (10)	0.0298 (11)	0.0314 (11)	−0.0001 (8)	0.0051 (8)	−0.0043 (9)
C17	0.0373 (12)	0.0359 (12)	0.0320 (12)	0.0035 (10)	0.0070 (9)	0.0021 (10)
C18	0.0429 (13)	0.0526 (16)	0.0344 (12)	0.0052 (12)	0.0145 (10)	−0.0043 (12)
C19	0.0304 (11)	0.0468 (15)	0.0442 (14)	0.0054 (10)	0.0105 (10)	−0.0116 (12)
C20	0.0338 (12)	0.0358 (13)	0.0432 (14)	0.0078 (10)	0.0040 (10)	−0.0038 (11)
C21	0.0340 (11)	0.0343 (12)	0.0305 (12)	0.0038 (10)	0.0087 (9)	0.0003 (10)

### Geometric parameters (Å, °)

**Table:**

Cl1—C2	1.743 (2)	N13—H13B	0.9100
C2—C3	1.375 (4)	N13—H13C	0.9100
C2—C7	1.378 (4)	C14—C15	1.530 (3)
C3—C4	1.383 (3)	C14—H14A	0.9800
C3—H3A	0.9500	C14—H14B	0.9800
C4—C5	1.384 (3)	C14—H14C	0.9800
C4—H4A	0.9500	C15—C16	1.512 (3)
C5—C6	1.385 (3)	C15—H15A	1.0000
C5—C8	1.518 (3)	C16—C17	1.381 (3)
C6—C7	1.387 (3)	C16—C21	1.393 (3)
C6—H6A	0.9500	C17—C18	1.390 (3)
C7—H7A	0.9500	C17—H17A	0.9500
C8—O9	1.425 (2)	C18—C19	1.382 (4)
C8—C10	1.520 (3)	C18—H18A	0.9500
C8—H8A	1.0000	C19—C20	1.383 (3)
O9—H9A	0.8400	C19—H19A	0.9500
C10—O11	1.246 (3)	C20—C21	1.382 (3)
C10—O12	1.267 (2)	C20—H20A	0.9500
N13—C15	1.503 (3)	C21—H21A	0.9500
N13—H13A	0.9100		
			
C3—C2—C7	121.1 (2)	H13A—N13—H13C	109.5
C3—C2—Cl1	119.5 (2)	H13B—N13—H13C	109.5
C7—C2—Cl1	119.4 (2)	C15—C14—H14A	109.5
C2—C3—C4	119.1 (2)	C15—C14—H14B	109.5
C2—C3—H3A	120.5	H14A—C14—H14B	109.5
C4—C3—H3A	120.5	C15—C14—H14C	109.5
C3—C4—C5	121.3 (2)	H14A—C14—H14C	109.5
C3—C4—H4A	119.3	H14B—C14—H14C	109.5
C5—C4—H4A	119.3	N13—C15—C16	110.97 (17)
C4—C5—C6	118.5 (2)	N13—C15—C14	108.59 (18)
C4—C5—C8	118.86 (19)	C16—C15—C14	112.37 (19)
C6—C5—C8	122.64 (19)	N13—C15—H15A	108.3
C5—C6—C7	121.0 (2)	C16—C15—H15A	108.3
C5—C6—H6A	119.5	C14—C15—H15A	108.3
C7—C6—H6A	119.5	C17—C16—C21	118.5 (2)
C2—C7—C6	119.1 (2)	C17—C16—C15	119.8 (2)
C2—C7—H7A	120.4	C21—C16—C15	121.69 (19)
C6—C7—H7A	120.4	C16—C17—C18	121.0 (2)
O9—C8—C5	110.47 (15)	C16—C17—H17A	119.5
O9—C8—C10	108.78 (16)	C18—C17—H17A	119.5
C5—C8—C10	112.68 (16)	C19—C18—C17	119.6 (2)
O9—C8—H8A	108.3	C19—C18—H18A	120.2
C5—C8—H8A	108.3	C17—C18—H18A	120.2
C10—C8—H8A	108.3	C18—C19—C20	120.1 (2)
C8—O9—H9A	109.5	C18—C19—H19A	120.0
O11—C10—O12	125.27 (19)	C20—C19—H19A	120.0
O11—C10—C8	119.05 (17)	C21—C20—C19	119.8 (2)
O12—C10—C8	115.68 (18)	C21—C20—H20A	120.1
C15—N13—H13A	109.5	C19—C20—H20A	120.1
C15—N13—H13B	109.5	C20—C21—C16	121.0 (2)
H13A—N13—H13B	109.5	C20—C21—H21A	119.5
C15—N13—H13C	109.5	C16—C21—H21A	119.5
			
C7—C2—C3—C4	−0.5 (4)	C5—C8—C10—O11	98.5 (2)
Cl1—C2—C3—C4	176.8 (2)	O9—C8—C10—O12	155.95 (17)
C2—C3—C4—C5	−0.1 (4)	C5—C8—C10—O12	−81.2 (2)
C3—C4—C5—C6	0.4 (3)	N13—C15—C16—C17	−139.6 (2)
C3—C4—C5—C8	178.0 (2)	C14—C15—C16—C17	98.6 (2)
C4—C5—C6—C7	−0.2 (3)	N13—C15—C16—C21	42.8 (3)
C8—C5—C6—C7	−177.7 (2)	C14—C15—C16—C21	−79.0 (3)
C3—C2—C7—C6	0.8 (4)	C21—C16—C17—C18	0.9 (3)
Cl1—C2—C7—C6	−176.52 (19)	C15—C16—C17—C18	−176.8 (2)
C5—C6—C7—C2	−0.4 (3)	C16—C17—C18—C19	−1.3 (4)
C4—C5—C8—O9	−81.8 (2)	C17—C18—C19—C20	0.5 (4)
C6—C5—C8—O9	95.7 (2)	C18—C19—C20—C21	0.8 (3)
C4—C5—C8—C10	156.32 (19)	C19—C20—C21—C16	−1.2 (3)
C6—C5—C8—C10	−26.2 (3)	C17—C16—C21—C20	0.4 (3)
O9—C8—C10—O11	−24.4 (3)	C15—C16—C21—C20	178.1 (2)

### Hydrogen-bond geometry (Å, °)

**Table:**

*D*—H···*A*	*D*—H	H···*A*	*D*···*A*	*D*—H···*A*
O9—H9A···O9^i^	0.84	2.26	2.939 (2)	139
O9—H9A···O11^i^	0.84	2.09	2.826 (2)	146
N13—H13A···O12^ii^	0.91	1.89	2.798 (2)	172
N13—H13B···O11	0.91	1.83	2.731 (2)	169
N13—H13C···O12^iii^	0.91	1.88	2.779 (2)	171

Symmetry codes: (i) −*x*+1, *y*+1/2, −*z*+1; (ii) *x*, *y*−1, *z*; (iii) −*x*+2, *y*−1/2, −*z*+1.
